# A New Framework for Precise Identification of Prostatic Adenocarcinoma

**DOI:** 10.3390/s22051848

**Published:** 2022-02-26

**Authors:** Sarah M. Ayyad, Mohamed A. Badawy, Mohamed Shehata, Ahmed Alksas, Ali Mahmoud, Mohamed Abou El-Ghar, Mohammed Ghazal, Moumen El-Melegy, Nahla B. Abdel-Hamid, Labib M. Labib, H. Arafat Ali, Ayman El-Baz

**Affiliations:** 1 Computers and Systems Department, Faculty of Engineering, Mansoura University, Mansoura 35511, Egypt; sarah_ayyad@mans.edu.eg (S.M.A.); nahla_bishri@mans.edu.eg (N.B.A.-H.); labib_essa@mans.edu.eg (L.M.L.); h.arafat_ali@mans.edu.eg (H.A.A.); 2 Radiology Department, Urology and Nephrology Center, Mansoura University, Mansoura 35516, Egypt; mohammed.ali.badawy@gmail.com (M.A.B.); maboelghar@mans.edu.eg (M.A.E.-G.); 3 BioImaging Laboratory, Bioengineering Department, University of Louisville, Louisville, KY 40292, USA; mnsheh01@louisville.edu (M.S.); ammost01@louisville.edu (A.A.); ahmahm01@louisville.edu (A.M.); 4 Department of Electrical and Computer Engineering, College of Engineering, Abu Dhabi University, Abu Dhabi 59911, United Arab Emirates; mohammed.ghazal@adu.ac.ae; 5 Department of Electrical Engineering, Assiut University, Assiut 71511, Egypt; moumen@aun.edu.eg; 6 Faulty of Artificial Intelligence, Delta University for Science and Technology, Mansoura 35516, Egypt

**Keywords:** prostate cancer, MRI, texture analysis, shape features, functional features, computer-aided diagnosis, PSA

## Abstract

Prostate cancer, which is also known as prostatic adenocarcinoma, is an unconstrained growth of epithelial cells in the prostate and has become one of the leading causes of cancer-related death worldwide. The survival of patients with prostate cancer relies on detection at an early, treatable stage. In this paper, we introduce a new comprehensive framework to precisely differentiate between malignant and benign prostate cancer. This framework proposes a noninvasive computer-aided diagnosis system that integrates two imaging modalities of MR (diffusion-weighted (DW) and T2-weighted (T2W)). For the first time, it utilizes the combination of functional features represented by apparent diffusion coefficient (ADC) maps estimated from DW-MRI for the whole prostate in combination with texture features with its first- and second-order representations, extracted from T2W-MRIs of the whole prostate, and shape features represented by spherical harmonics constructed for the lesion inside the prostate and integrated with PSA screening results. The dataset presented in the paper includes 80 biopsy confirmed patients, with a mean age of 65.7 years (43 benign prostatic hyperplasia, 37 prostatic carcinomas). Experiments were conducted using different well-known machine learning approaches including support vector machines (SVM), random forests (RF), decision trees (DT), and linear discriminant analysis (LDA) classification models to study the impact of different feature sets that lead to better identification of prostatic adenocarcinoma. Using a leave-one-out cross-validation approach, the diagnostic results obtained using the SVM classification model along with the combined feature set after applying feature selection (88.75% accuracy, 81.08% sensitivity, 95.35% specificity, and 0.8821 AUC) indicated that the system’s performance, after integrating and reducing different types of feature sets, obtained an enhanced diagnostic performance compared with each individual feature set and other machine learning classifiers. In addition, the developed diagnostic system provided consistent diagnostic performance using 10-fold and 5-fold cross-validation approaches, which confirms the reliability, generalization ability, and robustness of the developed system.

## 1. Introduction

In the United States (US), as well as worldwide, prostate cancer (PCa) is one of the most common male malignancies, and the second most common cancer type in the US with a death rate of about 2.4% among male patients [1,2]. It is considered a serious disease because of the danger of its metastasis into other parts of the body, such as the bladder, bones, and rectum. By 2030, it is expected that there will be up to 1.7 M PCa patients worldwide, with nearly half a million corresponding deaths each year [3]. Fortunately, early detection of PCa leads to better treatment and a lower mortality rate. Throughout this paper, PCa refers specifically to prostatic adenocarcinoma, the pathological subtype responsible for 99% of prostate malignancies.

Multiple screening and diagnostic tests are used to search for symptoms of prostate cancer including prostate-specific antigen (PSA) blood test [4], digital rectal examination (DRE) [5], needle biopsy [6], and magnetic resonance imaging (MRI) [7]. All these methods have recognized shortcomings. For instance, because PSA levels are measured in the blood, situations such as inflamed prostate can produce a high PSA value and may lead to treatments that are not needed [8,9]. In the DRE test, the physician checks the prostate manually to feel the surface of the prostate for regions of hardness. This approach can only identify peripheral zone tumors and cannot identify transitional zone and central zone tumors, or tumor regions that are too small to be felt [3,8]. Transrectal ultrasound (TRUS) guided biopsy [8] is the gold standard diagnostic technique, where the doctor takes a set of small tissue samples from the prostate to investigate under a microscope for cancerous cells. However, it is a painful and expensive procedure, and has adverse effects, such as bleeding and infection [8,10].

Over the past decade, prostate MRI has come to be widely used for cancer detection, especially to discover and locate intraprostatic lesions [11,12]. As a result, large numbers of MR examinations need to be processed. There is general consensus that the MRI submodalities best suited for examination of PCa include T2 weighted (T2W), dynamic contrast-enhanced (DCE), and diffusion-weighted (DW). T2W is the most common type of MRI that employs the transverse magnetization time T2 to create a grayscale image of the scanned area of the body [13]. The idea behind DW-MRI is that it generates images with contrast that reflects differences in the microscopic movement of water molecules within tissues [14]. It can distinguish between benign and suspicious prostatic lesions according to apparent diffusion coefficient (ADC) values from the signal intensity in images obtained using different *b*-values [7]. Several studies employed apparent diffusion coefficient (ADC) maps, which are quantitative maps calculated from DW-MRI, for PCa diagnosis [3,7,12]. Moreover, the acquisition of DW images does not involve injecting a human with a contrast agent, unlike DCE-MRI [15]. DW- and T2W-MRI have acquired popularity as non-invasive imaging techniques for detecting prostate cancer and may overcome many of the flaws of other methods [9]. It is worth mentioning that a few studies have tried to advanced modeling using intra-voxel incoherent motion (IVIM) MR imaging for PCa diagnosis [16]. IVIM emerged as a different approach for obtaining of perfusion information. Significant limitations of the IVIM analysis include the influence of the *b*-values used in the measurements and lack of standardization of calculation of IVIM parameters.

Over the last two decades, computer-aided diagnosis (CAD) has become a key technology in healthcare with the potential to enhance diagnosis and detection of diseases and then improvements in treatment [17,18,19]. Incorporating artificial intelligence (AI) into CAD systems can help clinicians avoid subjective decisions and reducing reading time. A typical AI-based CAD system takes MR images, locates the prostate, detects tumors within the prostate, and then classifies which of those tumors are likely to be malignant [11]. In recent years, abundant research studies on CAD systems were published employing a variety of AI techniques [12,13,15,17,18,19]. CAD systems employing AI can be largely classified into handcrafted feature-based CAD and deep learning-based CAD. Our proposed framework falls under the category of handcrafted feature-based CAD. Handcrafted feature-based CAD has attained more popularity in texture classification than deep learning-based techniques [20], owing to the fact that texture data tend to occupy much higher dimensional manifolds compared to object recognition data. Furthermore, deep learning techniques require a huge number of images for training models. Many of the effective CAD systems created for PCa use a group of handcrafted features that were applied for medical and non-medical images.

## 2. Related Works

There are many works of prostate cancer CAD systems in the literature. CADs that rely on handcrafted features have become popular in the medical image analysis field. For example, the work in [19] employed only 215 texture features extracted from T2W-MRI images and combines the prediction results of 11 classifiers. A noteworthy contribution was the work on many different texture features. This implies that their work investigates many features that have not been used before in common CAD systems. This work is limited in several aspects. For one, it examines the peripheral zone only. It also used the default parameter setting for each classifier. Moreover, only voxels within the malignant areas that are observable in T2W-MR images were considered cancerous voxels, this implies that voxels in the cancerous areas, which did not appear in T2W, were deemed normal. In [17], the authors introduced a CAD system based on texture, spatial, and intensity features extracted from DW (*b* = 2000 s/mm^2^), ADC, and T2W images on 244 patients. The total number of features was 17. They applied random forest (RF) classifier and compared their model to previous CAD models based on support vector machine (SVM) assessed on the same test data. However, their model has some limitations in that they use high *b*-value of 2000 s/mm^2^, where many institutes may not have the equipment to acquire such images. In addition, they used an endorectal coil MRI (ERC) when ERC is not available in all institutions.

A new voxel-based classification CAD system was proposed in [21], where each voxel in the prostate gland will be classified as normal or cancerous by the means of four modalities of MRI. Their goal was to produce a probabilistic map of cancer location in the prostate. They extracted a set of texture, intensity, edge, and anatomical features. These features were further decreased to provide a group of significant features that accurately detect malignancies. The random forest was chosen as their base classifier. However, this study is limited in that authors used a small cohort of patients (only 17 patients). Another machine learning (ML) framework was introduced in [22], where authors tested seven different classifiers to identify the classification model that most correctly differentiates between high-risk prostate cancer patients and low-risk patients. They used 55 texture features for both ADC and T2W images on 121 patients.

Recently, authors in [9] created a model based on auto-fixed segmentation through identical VOIs automatically generated a spherical VOI with the center of the lesion image for quantifying the phenotype of clinically significant (CS) peripheral zone lesions. They used two different datasets and extracted 92 quantitative radiomics features. They showed that adding DCE-MR imaging features enhanced the AUC value from 0.81 to 0.87. This model has a limitation that is only applicable to peripheral zone lesions. In addition, many institutions are reluctant in applying contrast agent to the patients. Other researchers, such as those in [23], developed a predictive ML model based on manual segmentation of T2 images on 191 patients. They extracted 367 radiomic features including the features suggested by the radiologist. Moreover, they applied the maximum relevance minimum redundancy technique to elect a subset of correlated features. Four classifiers were applied to evaluate the model and the model was compared with radiologist assessments. The model is concerned with two tasks: (1) normal vs. cancerous prostate lesion and (2) clinically significant prostate cancer vs. clinically insignificant prostate cancer.

In recent years, the breakthrough of deep learning in the field of image processing has radically altered prostate cancer detection and grading using MRI images [24,25]. In the literature, different related attempts on PCa were published [13,26,27,28,29]. The prostate CAD in [13] deployed a fully automatic mono-parametric MRI malignant PCa identification and localization system, where authors proposed a new 3D sliding window technique, that preserved the 2D domain complexity while utilizing 3D information. Although there are available four different modalities in their public dataset, the authors used only the T2W sequence on 19 patients. A first attempt to produce probability maps for prostate cancer detection by applying deep learning was introduced in [26]. The authors enhanced the holistically nested edge detection (HED) deep CNN. The main advantage of their work was in collecting their dataset from six institutions worldwide. One limitation of the study, however, was that the selected patient cohorts comprised high and intermediate risk patients only. In the same context, the authors of [27] introduced another model that also utilized CNN to evaluate predefined regions on prostate MRI. Lesions were manually segmented by a radiologist. They used three independent cohorts to reduce overfitting for the neural network. A four-class CNN was trained using the fastai library. They utilized Cohen’s kappa to measure the agreement of the model with the radiologist and found a rather low agreement (kappa = 0.40) between the model-driven and radiologist scoring.

Recently, the authors of [28] introduced a new classification framework, which was trained using patient-level labels only applied for two datasets. Features extracted by employing seven 3D ResNet CNN architectures from DW images, T2W images, and ADC maps. Then, a two-level SVM classifier scheme was applied to integrate the selected feature vectors and normalized clinical features to obtain a final result of classification. However, there was a big difference in performance evaluation between radiologist and their CAD. Another recent study [29], proposed a Retina U-Net detection framework to locate the lesions and expected their most likely Gleason grade. They worked on both the lesion level and the patient level on two different datasets. On the lesion level, they reported a sensitivity of 100%, specificity of 79% and an AUC of 0.96 on the first dataset and a sensitivity of 100%, specificity of 80% and an AUC of 0.96 on the second dataset. However, at the patient level, they found a noticeably reduced performance on the first dataset (AUC = 0.87, sensitivity = 100%, and specificity = 37.5%) and on the second dataset as well (AUC = 0.91, sensitivity = 100%, and specificity = 76.2%). However, their model has two limitations. First, it needs additional validation to evaluate histological results of targeted biopsies to the lesions detected by the model. Second, the authors successfully trained their model on two different datasets, but it still performed differently with each of them. This shows that CAD for prostate MRI is a very challenging area of study. Table 1 reports a brief recapitulation of the reviewed CAD studies.

**Figure 1:**
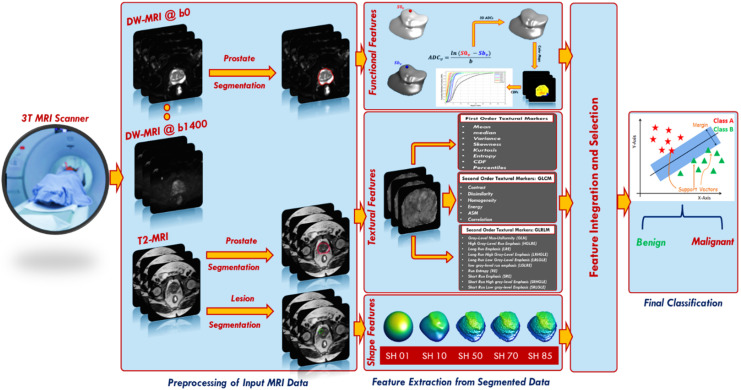
The proposed framework for early detection of prostatic adenocarcinoma.

To overcome the drawbacks of the aforementioned studies, we developed a new comprehensive framework (shown in Figure 1) for early identification of prostatic adenocarcinoma. The developed CAD system has the following main contributions: (i) it calculates functional features represented using cumulative distribution functions (CDFs) of 3D apparent diffusion coefficients (ADCs), estimated from segmented DW-MR images of the whole prostate. The proposed framework employs DW-MRI data gathered at nine different *b*-values (*b* = 0, 100, 200, 300, 400, 500, 600, 700, and 1400 s/mm^2^); thus, it is not sensitive to a specific choice of *b*-value and accounts for blood perfusion and water diffusion at both low and high *b*-values. (ii) The system extracts first and second order textural features that best describe the malignancy status of the prostate by applying novel rotation invariant techniques. (iii) It estimates best discriminating shape features by applying novel spherical harmonics analysis. (iv) Lastly, it integrates of functional, textural, shape features from two modalities of MRI (DW and T2W) with clinical biomarker (PSA) to produce a new comprehensive CAD system for the early identification of prostatic adenocarcinoma.

## 3. Material and Methods

The steps of the proposed framework are fully illustrated below and depicted in Figure 1.

### 3.1. Patient Population

A total of 92 patients, who were evaluated for prostate cancer, had undergone T2W-MR and DW-MR imaging at Urology and Nephrology Center, Mansoura University, Egypt, in the period between 2019 and 2021, were included. Inclusion criteria were: (a) high PSA (>4 ng/mL), (b) prostatic adenocarcinoma, and (c) physically and clinically fit for biopsy. Exclusion criteria were: (a) claustrophobia, (b) metallic implants and cardiac pace marker not suitable for MRI, or (c) abnormal coagulation. Twelve patients who had prostatitis and/or refused to participate in the study were then excluded. At the end, we were left with 80 patients with a mean age of 65.7 years, 43 diagnosed with benign prostatic hyperplasia, 37 with prostatic carcinomas). MRI imaging was performed on a 3T scanner (Ingenia, Philips Medical Systems, Best, Holland) with the following settings: number of signals averaging (NSA) = 4, flip angle = 90°, echo time (TE) = 88 ms, fasting imaging mode = echo planner imaging (EPI), and repetition time (TR) = 4734 ms, fat suppression = spectral attenuated inversion recovery (SPAIR), folder-over-suppression = over sampling. Our study was approved by the IRB (Code Number: R.21.04.1289), and all experiments were carried out according to the related rules and instructions, and all patients submitted informed consent to create the original dataset. An experienced radiologist with more than 10 years of hands-on-experience performed the manual segmentation of the prostate and all detected lesions using the Photoshop software tool.

### 3.2. Extracting Features

Extracting discriminative features from an area of interest is a fundamental characteristic of an efficient CAD system [8,30]. Common features for medical images include texture, topological, color, morphological, intensity, and various features [18,31,32]. Designing an efficient image feature set has a vital role in an accurate CAD system. In this framework, we extracted 266 image feature descriptors for the segmented regions of interest (ROIs) of lesion and prostate, and their extraction was primarily motivated by functional, textural, and morphological points of view. This implies that each voxel can be represented in a 266-dimensional feature space.

#### 3.2.1. Functional Features

**Figure 2:**
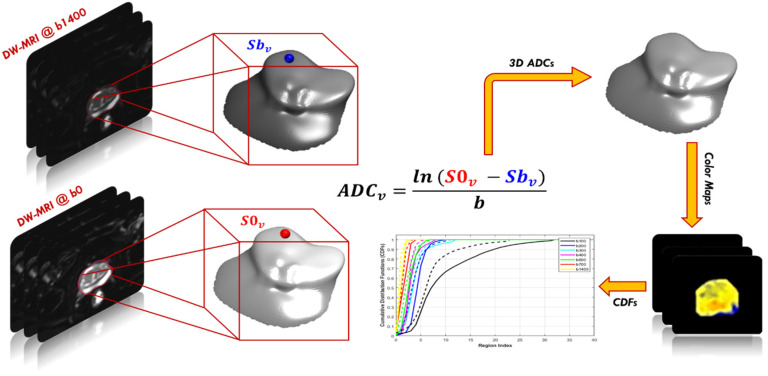
Calculations of voxel-wise apparent diffusion coefficients (ADC) for PCa and the cumulative distribution functions (CDFs) at different *b*-values from b100 to b1400.

**Figure 3:**
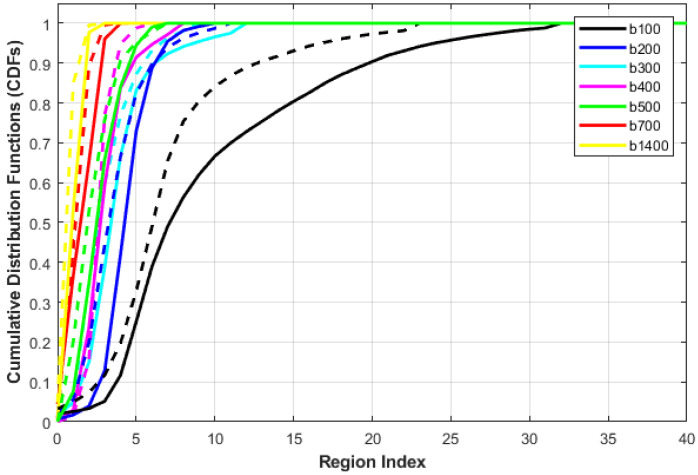
CDFs of ADC values for a benign case (solid) vs. a malignant case (dotted) for ADC maps obtained using different *b*-values from b100 to b1400. Note that region index indicates the different regions where the ADC values within the same range falls into.

Relying on the observations that the ADC measures the degree of molecular mobility and that tumors limit water motion because of the low permittivity of their cell membranes in comparison with the healthy tissue, a high-grade tumor has a smaller ADC value than a low-grade tumor, and a malignant tumor has a smaller ADC value than a benign one [33,34]. Hence, ADC maps could be used as discriminative functional features to enhance the diagnostic performance of PCa. To generate each ADC map, two DW-MR images are required; the first image is acquired at the baseline (*b*_0_), whereas the other image is acquired at a higher *b*-value. It is calculated at the voxel level as follows:

(1) $$ADC\left(x,y,z\right)=\frac{\ln\frac{s_{0}\left(x,y,z\right)}{s_{n}\left(x,y,z\right)}}{b_{n}-b_{0}}$$

where $s_{0}$ and $s_{n\;}$ are the signal intensity acquired at the baseline (*b* = 0) and a higher *b*-value *b_n_* ∈ {100, 200, 300, 400, 500, 600, 700, 1400} s/mm^2^, for the voxel at position x,y,z. Then, ADC maps were calculated at eight different *b*-values for all cases. Yet applying the voxel-wise ADCs of the whole prostate volume as discriminatory features faces two challenges. The main challenge is the data truncation or zero padding for small or bigger prostate volumes, because of the variable size of input data. The other challenge is that the needed training time to classify large data volumes is very high. We avoided these challenges through constructing the cumulative distribution functions (CDFs) of the 3D ADC maps at different *b*-values for each case. The smallest and largest ADCs are estimated for all datasets. After that, the ADCs are split into one hundred steps, so that all ADC values are maintained as coherent with a unified size without missing any information. Finally, CDFs of the 3D ADC map at different *b*-values are created and employed as discriminative features. Figure 2 shows the different steps to extract the functional features and Figure 3 shows an illustration for the estimated CDF of two cases (benign and malignant).

#### 3.2.2. Texture Features

**Figure 4:**
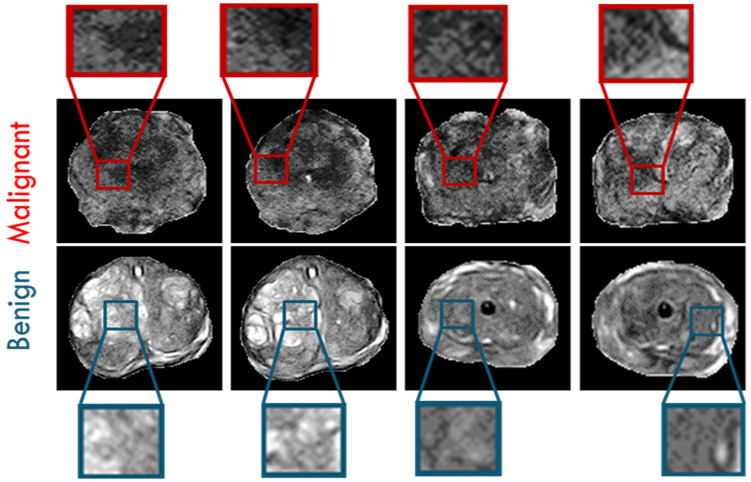
Illustrative examples of prostatic texture differences showing high gray level heterogeneity in four different malignant cases (first row) and low gray level heterogeneity in four different benign cases (second row).

Texture analysis (TA) is a significant field of study in medical imaging applications. TA has been utilized in the diagnosis of a variety of cancers [31,35,36,37]. There is no precise definition of TA, however, it can be described as the analysis of the spatial distribution of patterns that gives the visual appearance of roughness, randomness, smoothness, fluency, etc. [36]. It has been proven that MR images have various textural patterns that are often invisible to the human eye. Accordingly, texture analysis methods were utilized in our framework on the segmented region of interests (ROIs) of the whole prostate gland to precisely extract first and second order texture features that best characterize the homogeneity and heterogeneity between benign and malignant carcinomas. Usage of such extensive texture features depends on the fact that malignant carcinoma usually has high textural heterogeneity when compared to benign carcinoma. Figure 4 shows an illustrative example to compare benign cases and different grades of malignant cases in terms of texture differences.

Statistical TA methods can be largely classified into first-order and second-order texture features, based on the manner the gray levels are distributed over the pixels as follows.

**Figure 5:**
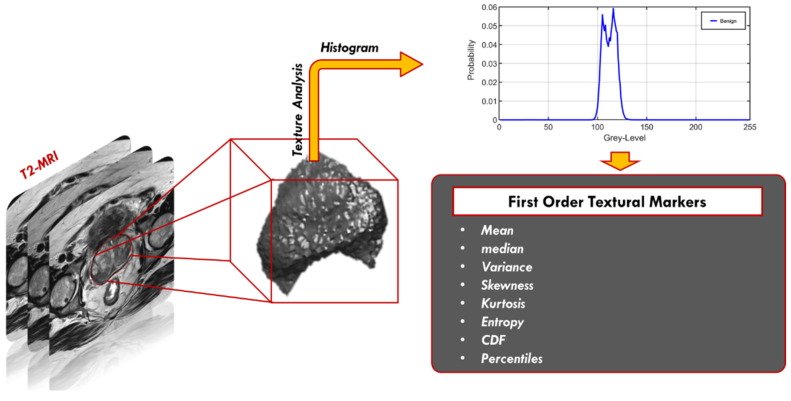
First-order textural features extraction.

First-order texture features: These texture features investigate the frequency distribution in the ROI through a histogram. Specifically, mean, median, variance, standard deviation, kurtosis, skewness, entropy, CDFs (N = 10), descriptive (mean, variance, Nobs, kurtosis), the number of points in each bin, size of bins, lower limit, bin width, cumulative frequency, and the gray level percentiles were calculated; from the 10th to the 100th percentiles with a step of 10%. A total of 36 first-order textural features were calculated. These features do not depend on the pixel’s location nor on the gray levels of other pixels in its immediate neighborhood (Figure 5).

**Figure 6:**
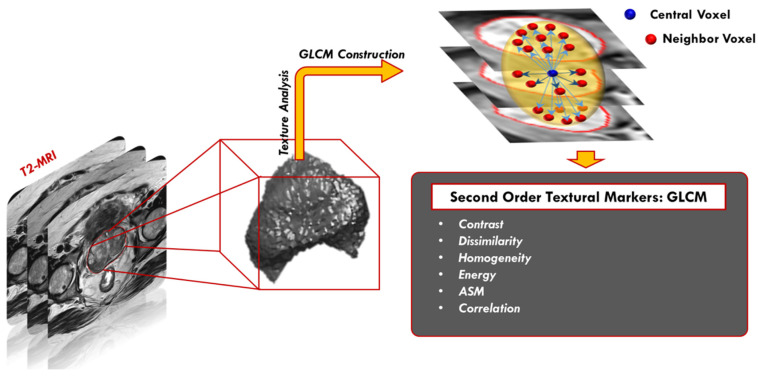
Second-order GLCM textural features extraction, where the central voxel of interest is shown in blue and the 26-neighbors are shown in red. The spatial relationship in the neighborhood block is obtained at different angles of zero, $\frac{\pi }{2}$, $\frac{\pi }{4}$, and $\frac{3\pi }{4}$.

Second-order texture features: These features depend on the probability that a pair of gray levels are selected at random distances and directions over the whole image [37]. In our framework, we used the gray level co-occurrence matrix (GLCM) [38] and gray level run length matrix (GLRLM) [39] as follows:

GLCM is based on computing how many times pairs of pixels with similar values and in a specified spatial relationship happen in a prostate object. The process starts with identifying the range of the original gray level of the prostate object and tumor object and normalizing these gray values to be in the range of 0 to 255 bringing on a GLCM matrix with a dimension of 256 × 256. After that, all possible pair combinations are specified to constitute the GLCM columns and rows. Finally, the value of each element within the matrix is estimated by determining the differences between each voxel and its neighbors. The neighborhood block is defined by a distance and an angle (the next voxel with the specified angle). Our neighborhood block has a distance of 1 in the Z-Plane (between different layers) and 1 in the XY plane (within the same layer). We worked with angles of zero, $\frac{\pi }{2}$, $\frac{\pi }{4}$, and $\frac{3\pi }{4}$. Thus, each voxel in our 3D object has a total of 26 neighbors (8 in the same layer, 9 in the upper layer and 9 in the lower layer). After creating the GLCM, it is normalized to have a sum of one to have the ability to extract the texture features depending on it. After each stage, a number of representative texture features (N = 6), specifically; contrast, correlation, angular second moment (ASM), dissimilarity, homogeneity, and energy were extracted as a summary of the GLCM (Figure 6).

**Figure 7:**
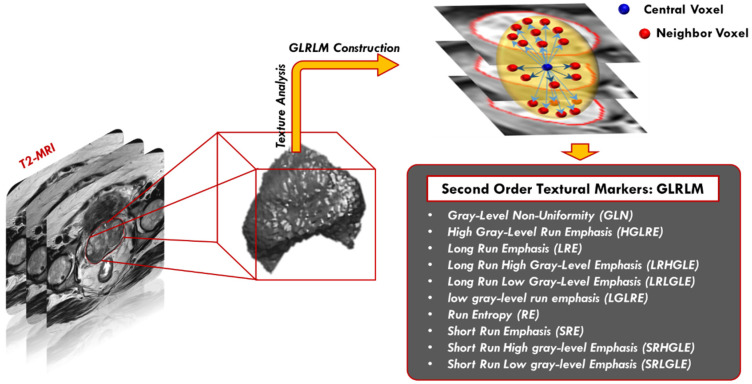
Second-order GLRLM textural features extraction, where the central voxel of interest is shown in blue and the 26-neighbors are shown in red. The spatial relationship is investigated to detect groups of sequential horizontal or vertical voxels with the same gray level.

GLRLM can be expressed as a set of pixels in a specific orientation having the same intensity value [40]. The number of such pixels specifies the length of the gray level run. GLRLM is a two-dimensional matrix, where each item p(i,j|θ) represents the number of elements (j) having an intensity (i). The normalized gray level matrix in our system has 256 rows with different numbers of columns between our objects. Herein, we searched for groups of sequential horizontal voxels in the XY plane and searched for vertical groups of voxels in the Z plane. Next, we estimated 16 features of the GLRLM, specifically: gray level non-uniformity, gray level non-uniformity normalized, high gray level run emphasis, gray level variance, long run emphasis, long run high gray level emphasis, long run low gray level emphasis, low gray level run emphasis, run length non-uniformity, run entropy, run length non-uniformity normalized, run variance, run percentage, short run emphasis, short run low gray level emphasis, and short run high gray level emphasis (Figure 7).

#### 3.2.3. Shape Features

**Figure 8:**
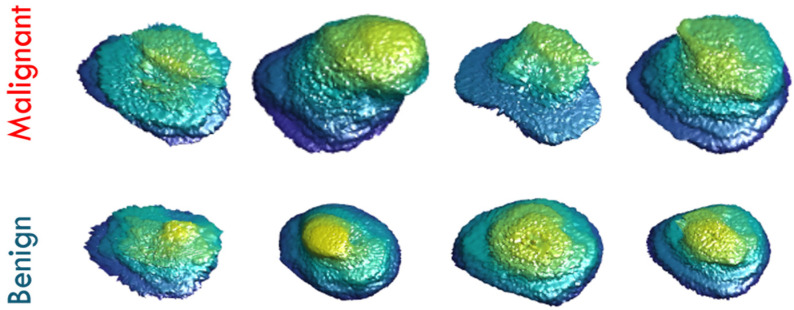
Visualization 3D shape differences between four malignant cases in the first row, and four benign cases in the second row.

For prostate cancer diagnosis and classification using T2W images, radiologists and researchers agree that morphological features are significant [23,41,42]. In the proposed framework, a number of shape features (morphological features) were also calculated to depict the morphology of the tumor candidate region. Shape features are extracted by capturing the structural information for the segmented region of interest of the lesion. The motivation for using shape features in our framework is based on the hypothesis that benign lesion has a less complex shape and a smaller growth rate than the malignant lesion. Figure 8 shows an illustrative example to compare between benign cases and malignant cases in terms of shape differences. In our work, we utilized the spectral spherical harmonics (SH) analysis [43] for extracting shape features for PCa diagnosis. A random point inside the region, or precisely in the interior of its convex kernel, was chosen to be the origin point (0, 0, 0). In this coordinate system, the surface of the region can be deemed a function of the polar and azimuth angle, f(θ,φ), which can be expressed as a linear set of base functions Y_τβ_ specified in the unit sphere. The modeling of spherical harmonics constructs a triangulated mesh approximating the surface of the lesion, afterwards maps it to the unit sphere by employing the attraction–repulsion technique [44].

Each cycle (*α*) of the attraction–repulsion approach operates as follows. Assume that $C_{\alpha ,i}$ represents the coordinates of the node on the unit sphere corresponding to mesh vertex *i* at the beginning of cycle *α*. Let $d_{\alpha ,ji}=C_{\alpha ,j}-C_{\alpha ,i}$ represent the vector from node *i* to node *j*; then, the Euclidean distance between nodes *i* and *j* is $d_{\alpha ,ji}=\;||d_{\alpha ,ji}||$. Assume that $J_{i}$ represents the index group of neighbors of vertex *i* in the triangulated mesh. Next, the attraction step updates the node’s locations to maintain it in the center with its neighbors according to Equation (2).

(2) $$C_{\alpha +1,i}^{\prime }=C_{\alpha ,i}+C_{A,1}\;\underset{j\epsilon J_{i}}{\sum }\left(d_{\alpha ,ji}\;d_{\alpha ,ji}^{2}+C_{A,2}\frac{d_{\alpha ,ji}}{d_{\alpha ,ji}}\right)\;$$

where $C_{A,1}$ and $C_{A,2}$ are parameters which specify the strength of the attractive force, j=1,2,3,…, J−1, J, and i=1, 2, 3,…, I−1, I. After that, the repulsion step enlarges the spherical mesh to hinder it from deteriorating according to Equation (3).

(3) $$C_{\alpha +1,i}^{\prime \prime }=C_{\alpha +1,i}^{\prime }+\frac{C_{R}}{2I}\;\overset{I}{\underset{j=1;j\neq i}{\sum }}\frac{d_{\alpha ,ji}}{d_{\alpha ,ji}^{2}}\;$$

where $C_{R}$ is a repulsion parameter that determines the shift incurred due to each other surface node and maintains a balance between the processing time and the accuracy. A small value of $C_{R}$ (e.g., 0.3 ≤$\;C_{R}\;$≥ 0.7) maintains a higher accuracy at the cost of increasing processing time. After that, the nodes are projected back onto the unit sphere through giving them the unit norm, and these are their coordinates at the start of the subsequent cycle according to Equation (4).

(4) $$C_{\alpha +1,i}=\frac{C_{\alpha +1,i}^{\prime \prime }}{∥C_{\alpha +1,i}^{\prime \prime }∥\;}\;$$

**Figure 9:**
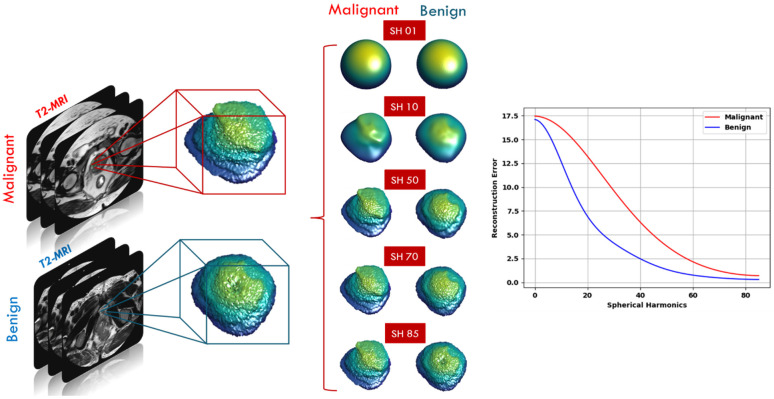
Reconstruction errors differences at different spherical harmonics (SH 01, 10, 50, 70, 85) between a malignant case and a benign case.

In the final cycle, $\alpha_{f}\;$, of the attraction–repulsion approach, the surface of the lesion is in a one-to-one correspondence with the unit sphere. Every point $C_{i}=\left(x_{i},\;y_{i},\;z_{i}\right)$ of the initial mesh has been mapped to a corresponding point $C_{\alpha_{f},i}=\left(\sin\theta_{i}\;\cos\varphi_{i},\;\sin\theta_{i}\sin\varphi_{i},\;\cos\theta_{i}\right)$ with polar angle $\theta_{i}\in \left[0,\;\pi \right]\;$ and azimuth angle $\varphi_{i}\in \left[0,\;2\pi \right]$. At this time, it is possibly to represent the lesion by a spherical harmonics series ($Y_{\tau \beta }$). Generating SH series is through solving an equation of isotropic heat for the surface that is considered a function on the unit sphere. The $Y_{\tau \beta }$ of degree τ with order β is identified according to Equation (5).

(5) $$Y_{\tau \beta }=\{\begin{array}{l} c_{\tau \beta }\;G_{\tau }^{\left|\beta \right|}\;\cos\theta \;\sin\left(\left|\beta \right|\varphi \right)\;-\tau \;\leq \;\beta \;\geq -1\\ \frac{c_{\tau \beta }}{\sqrt{2}}\;G_{\tau }^{\left|\beta \right|}\cos\theta \;\beta =0\\ c_{\tau \beta }\;G_{\tau }^{\left|\beta \right|}\;\cos\theta \;\cos\left(\left|\beta \right|\varphi \right)\;1\leq \;\beta \;\geq \;\tau \; \end{array}$$

where $c_{\tau \beta }$ is the spherical harmonics factor and $G_{\tau }^{\left|\beta \right|}$ represents the relevant Legendre polynomial of degree τ with order β. Benign lesions are described by a lower-order integration of spherical harmonic series, since their shapes are homogenous and less complex, whilst malignant lesions are described by a higher-order integration of spherical harmonic series since their shapes are heterogeneous and more complex. Subsequently, the overall number of markers measuring the shape complexity of the identified lesions is the number of the spherical harmonics. In our framework, a total number of 85 is sufficient to properly rebuild any lesion shape. Figure 9 shows the reconstruction errors differences at different harmonics between a malignant and a benign case.

### 3.3. Feature Integration and Selection

After functional and texture feature extraction from whole prostate gland, and shape feature extraction from lesion part, the features were integrated with the PSA clinical biomarker to produce a combined feature set (FS5) to be used for precise identification of prostatic adenocarcinoma.

A feature selection method generally aims to select the best features subset for correctly classifying objects to different classes in the dataset. Hence, an effective selection method of relevant and redundant features for PCa classification is required to increase classification accuracy, precision, and to minimize complexity. Many feature selection techniques have been developed in the domain of ML. They can be generally categorized into three approaches: filter, wrapper, and embedded [45,46,47,48,49]. A wrapper approach is applied in this framework. Generally, the wrapper-based feature selection approach uses learning procedures to determine which features are beneficial. It follows a greedy search strategy through the space of possible markers. In this study, we performed a bi-directional stepwise procedure [50] to find the optimal set of markers while taking into consideration the dependencies between features.

A bi-directional stepwise procedure is a combination of forward selection and backward elimination. As with forward selection, the procedure starts with no features and adds features using a pre-specified criterion. After adding each new feature, remove any features that no longer provide an improvement in the model fit (like backward selection). We applied the bi-directional algorithm with two thresholds of significance (0.05 and 0.1) on the combined feature sets (FS5) to obtain FS6 and FS7, respectively. A summary of the different feature sets is shown in Table 2.

### 3.4. Cancer Classification

Following feature extraction and feature selection, our framework proceeds with a classification stage to classify the input images as either benign tumors or malignant tumors. In the training stage, we used four different machine learning classifiers to attain the best possible results (e.g., support vector machine (SVM) [51], random forest (RF) [52], the C4.5 decision tree algorithm (DT) [53] and linear discriminant analysis (LDA) [54]). We chose these classifiers because of their popularity and strength in CAD systems. SVM is a kernel-based learner that is robust regarding the sparsity of data. RF has been highly successful as a general-purpose classifier and is considered as an ensemble of decision trees. DT is fairly robust in the presence of noise and most effective in CAD systems. On the other hand, we used the LDA classifier that permits the fast processing of data samples.

To better highlight the advantage of the feature integration process, we first assessed the performance of each feature set separately. Then, the individual feature sets are combined using a concatenation way, and utilized the aforementioned classifiers to get the final diagnosis. It should be noted that, for each classifier, a grid search algorithm was used to search for the optimal parameters, with the classification accuracy as the improvement criterion, for each of the classifier techniques being tested. In the Section 4, more details about the performance of feature integration will be provided.

### 3.5. Performance Evaluation

**Figure 10:**
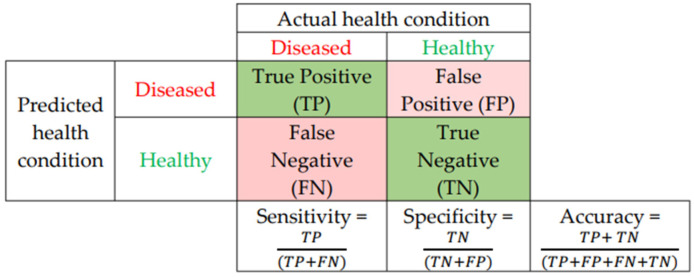
Performance metrics for evaluation of the proposed framework.

The new framework was tested on the datasets mentioned in Section 3.1. Performance evaluation of the new framework was performed using four accuracy metrics: (I) specificity, (II) sensitivity, (III) accuracy, and (IV) AUC. More details about these metrics can be found in Figure 10. For assessing the performance of the proposed framework, K-fold cross-validation was implemented for numerical evaluation. Three validation procedures were implemented: 5-fold, 10-fold, and leave-one-out cross validation. In order to mitigate accuracy variations, all the experiments were executed 10 times and the mean and standard deviation for the accuracy, sensitivity, specificity, and AUC were calculated for each feature set.

To investigate the added value of our framework, the developed CAD system was also compared to the clinical diagnosis made by an expert radiologist (10 years of experience in prostate MRI) for each patient on the basis of the Prostate Imaging Reporting and Data System (PIRADS) version 2 [55]. PIRADS can be used as a decision support system for targeting suspicious lesions. A radiologist scores each suspicious lesion on a scale from 1 to 5. Table 3 shows the scores that compose the PIRADS score system and their meaning in terms of the risk of the cancer being clinically significant. The radiologist was blinded to the respective PSA levels and pathological classification of tumors. PIRADS uses a dominant MRI sequence, including T2W, DWI, and ADC images.

## 4. Experimental Results

In order to validate and better highlight the effectiveness of the proposed framework, we first evaluated the proposed CAD system using each individual feature set (descriptions of each feature set are shown in Table 2). Furthermore, we evaluated the proposed CAD system using the combined features after applying feature selection, resulting in a notably improved classification performance. All the seven feature sets were evaluated and compared their performance using SVM, RF, DT, and LDA classifiers. For the purpose of applying the grid search algorithm, we created models for various combinations of parameters, assess the model for each combination and saved the results. We considered the best sets of parameters for each classifier as follows: For SVM we choose the gaussian kernel function for FS2, FS4, and FS6, linear kernel for FS1, and polynomial kernel of order 2 for other feature sets. For RF we set the number of learning cycles to 30. For DT we used Gini diversity index as the split criterion, and the number of splits varied according to feature set (10 for FS1, FS5, FS6, and FS7, 1 for FS2 and FS4, and 4 for FS3). For LDA, the Discriminant type was assumed to be diagLinear for FS1 and FS2 and Linear to other feature sets.

Table 4, Table 5, Table 6 and Table 7 present the classification performance using SVM, RF, DT, or LDA classifier, respectively, under the three validation schemas. Overall, the obtained results showed that the performance based on feature set FS7 is much better than all other individual feature sets and this highlights the advantage of the features integration and selection process in the proposed framework. It also showed that using a significance threshold = 0.1 provides better results than using a significance threshold = 0.05. In the three validation schemas, the SVM classifier outperformed all other classifiers. Since SVM demonstrated the best diagnostic capabilities, it was selected for the proposed framework. SVM is also well-known for its great regularization capabilities preventing overfitting. In terms of assessing the individual feature sets, the best results were achieved reassuringly by the functional features (FS1) and this for almost all classifiers. As shown in Table 5, functional features achieved the best classification performance for all experiments running in 5-fold cross validation with 86.67% ± 1.56% of accuracy, 76.58% ± 1.27% of sensitivity, 95.35% ± 2.68% of specificity, and 0.8603% ± 0.0152% of AUC. The second-ranking performance was achieved by texture features (FS2). PSA alone attained the lowest performance.

It can be noted from comparing the performance metrics of the four classifiers for the different validation schemas, that we can find that there is a low variance between the results in the same classifier and this is a good indicator of a good fit model of ML. It should be pointed out that the implementation of *k*-fold cross-validation was guaranteed to achieve a balanced reduction of variance and bias in classifier performance estimation.

To highlight the advantages of the proposed feature set (FS7), we provided a summary of comparison between the different classifiers using the three validation schemas applied on FS7 only in Table 8. From this table, the best results were achieved for FS7 by the SVM classifier using leave-one-out cross validation with 88.57% ± 0.00% of accuracy, 81.08% ± 0.00% of sensitivity, 95.35% ± 0.00% of specificity, and 0.8821 ± 0.00 of AUC. The second highest performance results were also achieved for FS7 by the RF classifier using leave-one-out cross validation with 86.25% ± 1.48% of accuracy and 93.95% ± 2.59% of specificity, and 0.8564 ± 0.0141 of AUC, while the DT achieved the highest sensitivity of 83.78% ± 0.00%. This suggests that using SVM classifier for classification is a promising one.

In a clinical setting, the AUC value is grouped into three grades: (1) acceptable when the score ranges from 0.7 to 0.8, (2) excellent when the score ranges from 0.8 to 0.9, and (3) outstanding when the score is over 0.9 [21,49]. In this regard, the proposed framework upgrades the CAD system from an acceptable grade to an excellent grade using the proposed feature set (FS7). The AUC reached in leave-one-out cross validation using SVM classifier was an average of 0.88 with FS7, while it was an average of 0.87 with FS5. This confirms that the results provided here constitute strong evidence to support the proposed feature integration hypothesis and feature selection method.

**Figure 11:**
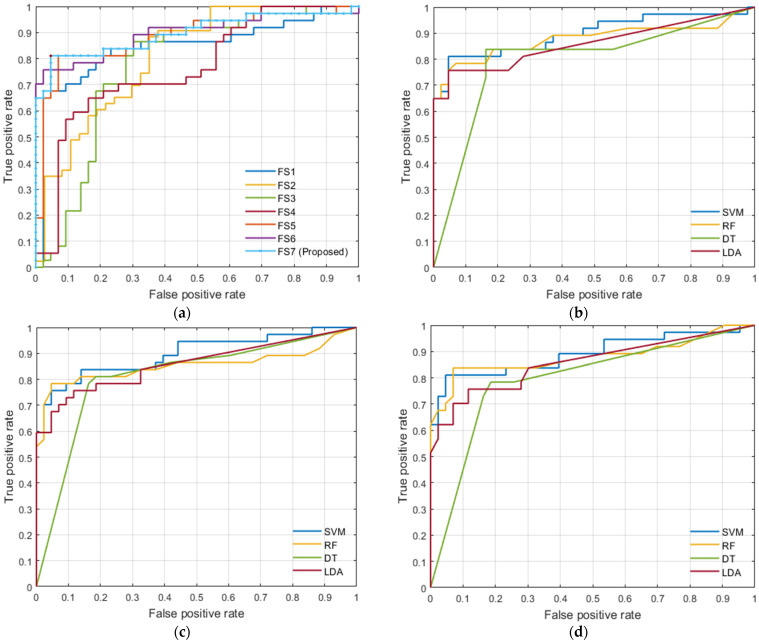
ROC curves of (**a**) SVM comparing various feature sets using leave-one-out cross validation, (**b**) different classifiers comparison using FS7 along with leave-one-out cross validation, (**c**) different classifiers comparison using FS7 along with 5-fold cross validation, and (**d**) comparison of classifiers using FS7 along with 10-fold cross validation.

The receiver operating characteristics (ROC) curves for SVM using leave-one-out cross validation for all feature sets and the proposed FS7 using the three validation schemas in all classifiers are visualized in Figure 11. ROC shows the trade-off between true positive rate (sensitivity) and false negative rate (1—specificity). As shown in this figure, the ROC area of the proposed FS7 is the maximum when compared to other feature sets and this highlights the advantages of using the proposed feature set (FS7) over other feature sets. Furthermore, the functional features demonstrated a potential in identifying the malignancy status of a given prostate tumor. Moreover, SVM classifier is optimal in comparison to other classifiers evidenced by the highest AUC, as shown in Figure 11.

The PIRADS v2 scores resulted in the correct classification of 47 of 51 lesions (17 benign prostatic hyperplasia cases and 30 prostatic carcinomas cases). The four lesions that were not detected were benign lesions. The counts of PIRADS 3 were 29 from 80 cases that were undecided and this is considered high, more specifically the number of PIRADS 3 was 22 for benign prostatic hyperplasia cases and 6 for prostatic carcinomas. For a fair comparison, we can say that PIRADS missed 33 cases and there is a large degree of subjectivity. It is worth mentioning that there were no cases given a score of PIRADS 1 by the radiologist as PIRADS 1 lesions were generally not biopsied and therefore are only partially included in this study. These results stress the need for our CAD system to distinguish these equivocal lesions further into insignificant and significant tumors and to be more objective.

## 5. Discussion

With a high mortality rate, prostate cancer is considered as one of the worldwide leading cancerous causes of death. Precisely detecting prostate cancer at early stages could enhance the survival opportunities of the patients.

In this study, we introduce a new comprehensive framework to precisely differentiate between malignant and benign prostate cancer. The classification results of the developed CAD system that combined different imaging markers and clinical biomarkers showed high accuracy, sensitivity, specificity, and AUC. These results revealed the feasibility and efficacy of the developed CAD system to non-invasively identify prostatic adenocarcinoma at an early stage. Classification results attained using individual features (functional or texture or shape or PSA) had lower accuracy, sensitivity, specificity, and AUC compared to using combined features. Validation schema experiments further reinforce the reliability of our accuracy findings.

It is worth noting that this high diagnostic accuracy of the developed CAD system is obtained by SVM classifier due to its ability to handle non-planar class boundaries in an efficient and stable manner by using kernels [51]. Throughout this study, we have extracted functional features from the whole prostate ROI, textural feature from the whole prostate ROI, and shape features from the lesion ROI only. This can be justified in part by the fact that the prostate gland as a whole is more informative in terms of functionality analysis to study the motion of water molecules quantified by 3D ADCs and in terms of texture analysis by providing a larger area to study the spatial relationship between the neighboring voxels using various first and second order texture features. On the other hand, lesion ROI is more informative in terms of the lesion shape, size, and complexity of the surface.

Most of the clinical research calculates the ADC at a few select *b*-values [9,14,17,26,27], typically one of the lower *b*-values and one of the higher *b*-values along with the baseline (*b* = 0). This study utilized nine different *b*-values to accurately differentiate between malignant and benign cases.

There are several other studies that designed to extract different features from DW MRI and T2 MRI that check the existence of PCa [9,17,21,23,42]. Few have investigated using clinical features for PCa detection [56]. To the best of our knowledge, there is no work in the literature that was conducted a fusion of texture, functional, shape imaging features and clinical features for PCa detection. This implies that our study could be a base for further studies using a combination of imagery and clinical MRI derived features to discriminate between benign and malignant PCa. A direct comparison between our study and other literature would not be objective as the other studies incorporate different data sets and variations in imaging protocols. However, our results are in line with the findings of other studies [9,17,21,23], showing that the combination of different features attained higher classification results than using textural features or morphological features alone. Moreover, our developed CAD system achieved AUC of 0.882, an improvement over the study done of Lemaitre et al. [21] that produced an AUC of 0.838, despite using more imaging modalities than used our study. Additionally, our performance is greater than the study done by Bleker et al. [9], as they attained an AUC of 0.838, in spite of using greater sample size in their study (206 patients).

Adding this superiority of the developed system (compared to the literature) to our experimental findings (Table 4, Table 5, Table 6, Table 7 and Table 8), reflect the accuracy of our methodology and the potential clinical utility of these provided approaches when used with MR imaging in computer-aided diagnosis of prostate cancers.

## 6. Conclusions

In this study, a new CAD system for the precise identification of prostate cancer in multiple modalities of MRI is presented. The paper depicts the complete design of the proposed framework to assess the potential role of integrating functional, textural, shape features combined with PSA and provides a detailed diagnostic performance analysis. The proposed framework achieved a high classification accuracy of 88.75%, a sensitivity of 81.08%, a specificity of 95.35%, an AUC of 0.8821, in differentiating benign tumor from malignant tumor using SVM along with the selected features set (FS7) outperforming the diagnostic abilities of individual and combined feature sets and other well-known ML classification models (e.g., LDA, RF, and DT). We have also included three validation schemas (5-fold, 10-fold, and leave-one-out cross validation). These results highlight the advantage of integrating clinical biomarker with DW-MRI and T2W-MRI for prostate cancer diagnosis.

Our framework is not devoid of limitations. Firstly, it needs manual lesion segmentation, that supposes that the tumor can be discovered and segmented accurately. Secondly, we studied DW-MRI and T2W-MRI acquired from one hospital and using only one type of scanner. The focus of the future work will be on validating our framework on a large dataset to verify the robustness of the proposed system. Further research is also needed to investigate whether we can reduce the number of *b*-values that are not informative enough. In addition, we plan to investigate the potential capabilities of the IVIM model in early and precise identification of prostatic adenocarcinoma. Moreover, a deep learning-based CAD will be established for the fully automated extraction of imagery features.

## Figures and Tables

**Table 1:** A brief comparison between previous prostate MRI CAD studies.

Reference	Year	Type of Approach	Features Type	Classes	Images Sequences	No. of Patients Involved	Accuracy Result
[17]	2017	Handcrafted features-based CAD	Spatial, intensity, and texture	Benign, Gleason 6, Gleason 7, Gleason 8, Gleason 9, Gleason 10	B2000, ADC, and T2W	224	SVM model achieved an AUC value of 0.86, while Random Forest achieved an AUC of 0.93
[19]	2016		Texture	Malignant or benign	T2W	45	It has a value of 0.93 AUC
[21]	2017		Texture, intensity, edge, and anatomical	Voxel-based classification	DWI, T2W, DCE, and MRSI	17	Classification performance of an average AUC of 0.836 ± 0.083 is achieved
[22]	2019		Texture	High risk patients and low risk patients	T2WI and ADC	121	Quadratic kernel based SVM is the best model with an accuracy of 0.92
[9]	2020		Texture and intensity	Benign and/or cs PCa vs. non-cs PCa	B50, b400, b800, b1400, T2WI, DCE, and ADC	206	It has an average AUC value of 0.838
[23]	2020		Shape, texture, and statistical texture	Normal vs. cancerous prostate lesion and clinically significant PCa vs. clinically insignificant PCa	ADC and T2WI	191	AUC value for normal vs. cancerous classification is 0.889, while the AUC value for clinically significant PCa vs. clinically insignificant PCa is 0.844
[13]	2019	Deep learning-based CAD		Produces a voxel probability map	T2WI	19	The model attained an AUC value of 0.995, a recall of 0.928, and an accuracy of 0.894.
[26]	2018			Produces probability maps to detect prostate cancer	T2WI, ADC, and high *b*-value (b1500 for cases imaged without ERC insertion, and b-2000 with ERC insertion)	186	The model attained an average AUC value of 0.94 in the peripheral zone and an average AUC value of 0.92 in transition zone.
[27]	2020			Gives a PI-RADS score to a lesion detected and segmented by a radiologist	T2WI, T1WI, ADC, and (b1500 or b2000)	687	Kappa = 0.40, sensitivity = 0.89, and specificity = 0.73.
[28]	2021			Probability that patient has prostate cancer	T2WI, b200, ADC in the first dataset, T2WI, ADC in the second dataset	249 patients in the 1st dataset and 282 patients in the 2nd dataset	AUC value for the first dataset was 0.79, and for the second dataset was 0.86.
[29]	2021			Predicting the Gleason grade group and classifying benign vs. csPCa	T1WI and T2WI	490 cases for training and 75 cases for testing from 2 different datasets	On the lesion level, AUC of 0.96 for both the first and second datasets. On the patient level, AUC of 0.87 and 0.91, for the first and second datasets, respectively.

**Table 2:** Details of the extracted feature sets. Let ST denote the significance threshold.

Feature Set No.	Representation	Number of Extracted Features
FS1	Functional features for whole prostate	122
FS2	Texture features for whole prostate	58
FS3	Shape features for lesion only	85
FS4	Prostatic-specific antigen (PSA)	1
FS5	Combined features (FS1 + FS2 + FS3 + FS4)	266
FS6	Selected features of FS5 with ST = 0.05	101
FS7-Proposed	Selected features of FS5 with ST = 0.1	162

**Table 3:** PIRADS scores.

PIRADS Score	Definition
1	Most probably benign (normal)
2	Probably benign tumor
3	Intermediate (the presence of clinically significant cancer is equivocal)
4	Probably malignant tumor
5	Most probably malignant tumor

**Table 4:** Comparison of experimental results of classification accuracy (%), sensitivity (%), specificity (%), and AUC (in terms of mean ± standard deviation) using the proposed SVM classification model, where 𝛜 indicates 1.0 × 10^−5^.

Feature Set	Validation	Accuracy	Sensitivity	Specificity	AUC
FS1	5-fold	81.81 ± 2.13	71.17 ± 3.6	90.96 ± 3.18	0.8106 ± 0.0215
	10-fold	83.75 ± 2.00	72.59 ± 2.25	93.35 ± 2.89	0.8297 ± 0.0197
	Leave-one-out	82.50 ± 𝛜	67.57 ± 𝛜	95.35 ± 𝛜	0.8146 ± 𝛜
FS2	5-fold	75.83 ± 1.72	61.26 ± 2.01	88.37 ± 3	0.7482 ± 0.0166
	10-fold	74.82 ± 2.26	61.39 ± 3.45	86.38± 2.3	0.7389 ± 0.0231
	Leave-one-out	77.50 ± 𝛜	64.86 ± 𝛜	88.37 ± 𝛜	0.7662 ± 𝛜
FS3	5-fold	74.28 ± 1.87	81.46 ± 2.25	68.11 ± 2.97	0.7479 ± 0.0183
	10-fold	74.58 ± 2.00	**80.63 ± 3.63**	69.38 ± 2.48	0.75 ± 0.0206
	Leave-one-out	77.50 ± 𝛜	86.49 ± 𝛜	69.77 ± 𝛜	0.7813 ± 𝛜
FS4	5-fold	72.50 ± 𝛜	51.35 ± 𝛜	90.70 ± 𝛜	0.7102 ± 𝛜
	10-fold	72.50 ± 𝛜	51.35 ± 𝛜	90.70 ± 𝛜	0.7102 ± 𝛜
	Leave-one-out	72.50 ± 𝛜	51.35 ± 𝛜	90.70 ± 𝛜	0.7102 ± 𝛜
FS5	5-fold	84.37 ± 2.01	75.23 ± 4.25	92.25 ± 2.57	0.8373 ± 0.021
	10-fold	84.50 ± 1.27	76.49 ± 2.72	91.39 ± 2.56	0.8394 ± 0.0127
	Leave-one-out	87.50 ± 𝛜	81.08 ± 𝛜	93.02 ± 𝛜	0.8705 ± 𝛜
FS6	5-fold	**85.42 ± 0.93**	73.87 ± 1.28	**95.35 ± 1.34**	0.8461 ± 0.0092
	10-fold	85.94 ± 0.83	74.33 ± 1.36	**95.93 ± 1.00**	0.8513 ± 0.0084
	Leave-one-out	86.25 ± 𝛜	75.68 ± 𝛜	95.35 ± 𝛜	0.8551 ± 𝛜
**FS7**	**5-fold**	85.18 ± 1.04	**78.38 ± 1.44**	91.03 ± 1.49	**0.8471 ± 0.0103**
	**10-fold**	**87.63 ± 1.53**	80.27 ± 2.11	93.95 ± 1.54	**0.8711 ± 0.0155**
	**Leave-one-out**	**88.75 ±** 𝛜	**81.08 ±** 𝛜	**95.35 ±** 𝛜	**0.8821 ±** 𝛜

**Table 5:** Comparison of experimental results of classification accuracy (%), sensitivity (%), specificity (%), and AUC (in terms of mean ± standard deviation) using a RF classification model, where 𝛜 indicates 1.0 × 10^−5^.

Feature Set	Validation	Accuracy	Sensitivity	Specificity	AUC
FS1	5-fold	86.67 ± 1.56	76.58 ± 1.27	95.35 ± 2.68	0.8603 ± 0.0152
	10-fold	86.09 ± 1.59	77.03 ± 1.35	93.9 ± 2.31	0.8546 ± 0.0154
	Leave-one-out	85.78 ± 1.24	76.35 ± 1.79	93.9 ± 2.83	0.8512 ± 0.0115
FS2	5-fold	76.25 ± 2.28	63.97 ± 4.03	86.82 ± 2.89	0.7539 ± 0.0234
	10-fold	76.67 ± 1.38	65.76 ± 4.03	86.05 ± 2.68	0.7591 ± 0.0148
	Leave-one-out	76.75 ± 1.00	65.4 ± 2.02	86.51 ± 3.08	0.7596 ± 0.0087
FS3	5-fold	73.25 ± 0.61	75.68 ± 1.71	71.16 ± 1.14	0.7342 ± 0.0065
	10-fold	72.68 ± 1.45	75.68 ± 1.45	70.1 ± 1.94	0.7289 ± 0.0153
	Leave-one-out	72.50 ± 𝛜	75.68 ± 𝛜	69.77 ± 𝛜	0.7272 ± 𝛜
FS4	5-fold	73.75 ± 𝛜	51.35 ± 𝛜	93.02 ± 𝛜	0.7219 ± 𝛜
	10-fold	73.57 ± 0.44	50.96 ± 0.94	93.02 ± 𝛜	0.72 ± 0.0047
	Leave-one-out	73.75 ± 𝛜	51.35 ± 𝛜	93.02 ± 𝛜	0.7219 ± 𝛜
FS5	5-fold	84.82 ± 1.82	77.22 ± 1.97	91.36 ± 2.05	0.8429 ± 0.0182
	10-fold	87.32 ± 1.56	79.54 ± 1.97	94.02 ± 1.69	0.8678 ± 0.0157
	Leave-one-out	86.13 ± 1.42	77.30 ± 1.32	93.72 ± 2.09	0.8551 ± 0.0138
FS6	5-fold	83.75 ± 0.95	75.29 ± 1.73	91.03 ± 2.30	0.8316 ± 0.0089
	10-fold	84.58 ± 1.56	76.58 ± 3.12	91.47 ± 1.09	0.8402 ± 0.0165
	Leave-one-out	86.38 ± 1.42	78.65 ± 2.24	93.02 ± 1.80	0.8584 ± 0.0144
FS7	5-fold	84.86 ± 1.5	77.78 ± 2.47	90.96 ± 2.31	0.8437 ± 0.015
	10-fold	85.63 ± 1.53	77.67 ± 1.31	92.73 ± 2.44	0.8505 ± 0.0147
	Leave-one-out	86.25 ± 1.48	77.30 ± 1.32	93.95 ± 2.59	0.8564 ± 0.0141

**Table 6:** Comparison of experimental results of classification accuracy (%), sensitivity (%), specificity (%), and AUC (in terms of mean ± standard deviation) using a DT classification model, where 𝛜 indicates 1.0 × 10^−5^.

Feature Set	Validation	Accuracy	Sensitivity	Specificity	AUC
FS1	5-fold	75.45 ± 2.86	76.35 ± 4.22	84.71 ± 6.62	0.7553 ± 0.0266
	10-fold	75.50 ± 1.27	77.30 ± 1.32	73.95 ± 1.74	0.7563 ± 0.0125
	Leave-one-out	77.50 ± 𝛜	72.97 ± 𝛜	81.40 ± 𝛜	0.7718 ± 𝛜
FS2	5-fold	70.63 ± 1.88	53.60 ± 4.25	85.27 ± 4.58	0.6944 ± 0.0182
	10-fold	71.00 ± 0.94	54.59 ± 3.15	85.12 ± 3.78	0.6978 ± 0.0082
	Leave-one-out	70.00 ± 𝛜	45.95 ± 𝛜	90.70 ± 𝛜	0.6832 ± 𝛜
FS3	5-fold	66.79 ± 1.13	61.78 ± 4.88	71.10 ± 3.90	0.6644 ± 0.0122
	10-fold	65.00 ± 2.85	62.16 ± 2.96	67.44 ± 3.89	0.6480 ± 0.0280
	Leave-one-out	66.25 ± 𝛜	70.27 ± 𝛜	62.79 ± 𝛜	0.6653 ± 𝛜
FS4	5-fold	66.88 ± 3.59	61.71 ± 2.88	71.32 ± 5.48	0.6652 ± 0.0347
	10-fold	67.50 ± 1.12	58.38 ± 2.76	75.35 ± 1.86	0.6686 ± 0.0117
	Leave-one-out	65.00 ± 𝛜	56.76 ± 𝛜	72.09 ± 𝛜	0.6442 ± 𝛜
FS5	5-fold	78.44 ± 2.32	79.39 ± 3.56	77.62 ± 4.93	0.7851 ± 0.0221
	10-fold	80.25 ± 3.10	79.46 ± 2.16	80.93 ± 5.58	0.8019 ± 0.0293
	Leave-one-out	82.50 ± 𝛜	83.78 ± 𝛜	81.40 ± 𝛜	0.8259 ± 𝛜
FS6	5-fold	79.84 ± 3.09	76.01 ± 4.95	83.14 ± 4.15	0.7958 ± 0.0312
	10-fold	79.82 ± 1.82	79.92 ± 4.30	79.73 ± 3.67	0.7983 ± 0.0185
	Leave-one-out	83.75 ± 𝛜	83.78 ± 𝛜	83.72 ± 𝛜	0.8375 ± 𝛜
FS7	5-fold	81.46 ± 1.97	77.93 ± 2.88	84.50 ± 3.72	0.8121 ± 0.019
	10-fold	80.36 ± 1.10	80.31 ± 2.78	80.40 ± 2.44	0.8035 ± 0.0112
	Leave-one-out	83.75 ± 𝛜	83.78 ± 𝛜	83.72 ± 𝛜	0.8375 ± 𝛜

**Table 7:** Comparison of experimental results of classification accuracy (%), sensitivity (%), specificity (%), and AUC (in terms of mean ± standard deviation) using an LDA classification model, where 𝛜 indicates 1.0 × 10^−5^.

Feature Set	Validation	Accuracy	Sensitivity	Specificity	AUC
FS1	5-fold	79.38 ± 0.88	72.97 ± 𝛜	84.88 ± 1.64	0.7893 ± 0.0082
	10-fold	79.75± 0.94	72.97 ± 𝛜	85.58 ± 1.74	0.7928 ± 0.0087
	Leave-one-out	80.00 ± 𝛜	72.97 ± 𝛜	86.05 ± 𝛜	0.7951 ± 𝛜
FS2	5-fold	73.03 ± 1.13	58.69 ± 1.89	85.38 ± 1.05	0.7203 ± 0.0116
	10-fold	72.92 ± 0.59	59.01 ± 1.86	84.89 ± 1.17	0.7195 ± 0.0064
	Leave-one-out	71.25 ± 𝛜	56.76 ± 𝛜	83.72 ± 𝛜	0.7024 ± 𝛜
FS3	5-fold	72.29 ± 0.86	74.33 ± 1.36	70.54 ± 2.19	0.7243 ± 0.0078
	10-fold	71.50 ± 1.22	74.6 ± 1.33	68.84 ± 1.86	0.7172 ± 0.0119
	Leave-one-out	72.50 ± 𝛜	75.68 ± 𝛜	69.77 ± 𝛜	0.7272 ± 𝛜
FS4	5-fold	73.13 ± 0.88	50 ± 1.91	93.02 ± 𝛜	0.7151 ± 0.0095
	10-fold	73.39 ± 0.56	50.58 ± 1.22	93.02 ± 𝛜	0.718 ± 0.0061
	Leave-one-out	73.75 ± 𝛜	51.35 ± 𝛜	93.02 ± 𝛜	0.7219 ± 𝛜
FS5	5-fold	81.56 ± 0.54	73.99 ± 1.31	88.08 ± 0.77	0.8103 ± 0.0057
	10-fold	81.75 ± 0.83	74.87 ± 1.24	87.67 ± 1.06	0.8127 ± 0.0083
	Leave-one-out	82.50 ± 𝛜	75.68 ± 𝛜	88.37 ± 𝛜	0.8202 ± 𝛜
FS6	5-fold	82.92 ± 0.59	73.42 ± 1.01	91.09 ± 0.86	0.8226 ± 0.0059
	10-fold	82.32 ± 0.8	72.97 ± 2.04	90.37 ± 0.82	0.8167 ± 0.0087
	Leave-one-out	82.50 ± 𝛜	72.97 ± 𝛜	90.70 ± 𝛜	0.8184 ± 𝛜
FS7	5-fold	83.00 ± 0.93	75.68 ± 𝛜	89.30 ± 1.54	0.8249 ± 0.0077
	10-fold	82.29 ± 0.47	75.68 ± 𝛜	87.98 ± 0.87	0.8183 ± 0.0043
	Leave-one-out	82.50 ± 𝛜	75.68 ± 𝛜	88.37 ± 𝛜	0.8202 ± 𝛜

**Table 8:** Comparison of experimental results of classification accuracy (%), sensitivity (%), specificity (%), and AUC (in terms of mean ± standard deviation) using the different classifiers for only our proposed feature set (FS7), where 𝛜 indicates 1.0 × 10^−5^.

Classifier	Validation	Accuracy	Sensitivity	Specificity	AUC
SVM	5-fold	**85.18 ± 1.04**	**78.38 ± 1.44**	**91.03 ± 1.49**	**0.8471 ± 0.0103**
	10-fold	**87.63 ± 1.53**	80.27 ± 2.11	**93.95 ± 1.54**	**0.8711 ± 0.0155**
	Leave-one-out	**88.75 ±** 𝛜	81.08 ± 𝛜	**95.35 ±** 𝛜	**0.8821 ±** 𝛜
RF	5-fold	84.86 ± 1.5	77.78 ± 2.47	90.96 ± 2.31	0.8437 ± 0.015
	10-fold	85.63 ± 1.53	77.67 ± 1.31	92.73 ± 2.44	0.8505 ± 0.0147
	Leave-one-out	86.25 ± 1.48	77.3 ± 1.32	93.95 ± 2.59	0.8564 ± 0.0141
DT	5-fold	81.46 ± 1.97	77.93 ± 2.88	84.50 ± 3.72	0.8121 ± 0.019
	10-fold	80.36 ± 1.1	**80.31 ± 2.78**	80.40 ± 2.44	0.8035 ± 0.0112
	Leave-one-out	83.75 ± 𝛜	**83.78 ±** 𝛜	83.72 ± 𝛜	0.8375 ± 𝛜
LDA	5-fold	83.00 ± 0.93	75.68 ± 𝛜	89.3 0± 1.54	0.8249 ± 0.0077
	10-fold	82.29 ± 0.47	75.68 ± 𝛜	87.98 ± 0.87	0.8183 ± 0.0043
	Leave-one-out	82.50 ± 𝛜	75.68 ± 𝛜	88.37 ± 𝛜	0.8202 ± 𝛜

## Data Availability

Data can be made available upon a reasonable request to the corresponding author.
